# Covered Self-Expanding Metal Stents Versus Multiple Plastic Stents for Benign Biliary Strictures: An Updated Meta-Analysis of Randomized Controlled Trials

**DOI:** 10.7759/cureus.24588

**Published:** 2022-04-29

**Authors:** Suprabhat Giri, Vaneet Jearth, Sridhar Sundaram

**Affiliations:** 1 Gastroenterology and Hepatology, Nizam's Institute of Medical Sciences, Hyderabad, IND; 2 Gastroenterology, Postgraduate Institute of Medical Education and Research, Chandigarh, IND; 3 Gastroenterology and Hepatology, Tata Memorial Hospital, Mumbai, IND

**Keywords:** meta-analysis, self-expanding metallic stent, plastic stent, ercp, benign biliary stricture

## Abstract

Optimal endoscopic management of benign biliary strictures (BBS) has been a matter of debate with choice of stent remaining largely at the discretion of the endoscopist. In this systematic review and meta-analysis, we compared self-expanding metal stents with multiple plastic stents for benign biliary strictures. A comprehensive search of literature from 2000 till September 2021 was done of various databases for randomized controlled trials evaluating stent placement for benign biliary strictures. Our primary aim was to compare outcomes of endoscopic therapy for BBS using covered self-expandable metal stents (cSEMS) and multiple plastic stents (MPS) in terms of stricture resolution, number of ERCP sessions, recurrence of stricture, stent migration, and moderate-severe adverse events. Eight randomized controlled trials (534 patients) were included in the meta-analysis. cSEMS were comparable to MPS for stricture resolution (risk ratio {RR}: 1.0, 95% CI: 0.89-1.08, p=1.00), recurrence of stricture (RR: 0.73, 95% CI: 0.35-1.53, p=0.13), stent migration (RR: 0.90, 95% CI: 0.54-1.52, p=0.26), and moderate-severe adverse events (RR: 1.04, 95% CI: 0.67-1.61, p=0.19) with low to moderate heterogeneity among studies. cSEMS required fewer sessions of ERCP for stricture resolution (mean difference: 1.88, 95% CI: 0.91-2.85, p<0.00001) but with significant heterogeneity among studies. No difference in stricture resolution was seen in subgroup analysis between anastomotic strictures, chronic pancreatitis, or bile duct injury. cSEMS are comparable to MPS in patients with benign biliary strictures in terms of stricture resolution, recurrence, and adverse effects, needing fewer sessions of ERCP. Larger studies comparing cost-effectiveness of cSEMS and MPS in BBS are needed.

## Introduction and background

Benign biliary strictures (BBS) occur due to various pancreaticobiliary inflammatory conditions like chronic pancreatitis (CP), primary sclerosing cholangitis and autoimmune pancreatitis, biliary anastomosis after liver transplant (LT), and post-operative bile duct injuries [1]. Depending on the site and extent of stenosis, BBS may be asymptomatic or present with symptoms of jaundice, abdominal pain with occasional life-threatening cholangitis. BBS is associated with long-term sequelae with an impact on liver function and the development of secondary biliary cirrhosis. Endoscopic therapy remains the mainstay in these patients with an aim to relieve symptoms of biliary obstruction, maintain drainage in the long term and preserve liver function [2].

Imaging is required prior to endoscopic management to differentiate benign from malignant strictures. Regular, symmetrical, short segment narrowing usually represents a BBS. On the other hand, irregular, asymmetrical strictures of long length (>14 mm) represent malignant strictures [3]. Tissue sampling using brush cytology or trans-papillary biopsy forceps is done to rule out malignancy. Endoscopic management is subsequently taken up with dilatation of BBS using a balloon or bougies [4]. One or more plastic stents and fully or partially covered self-expandable metal stents (cSEMS) are used to ensure stricture resolution [5]. While plastic stents need to be replaced at intervals of three months for up to 12 months, metal stents can be removed after an interval of six months to one year. Plastic stents are associated with a risk of biofilm development and stent occlusion. Metal stents, on the other hand, are associated with a risk of migration and cholecystitis [6]. Metal stents are also not useful in patients with hilar strictures, leading to placement across the biliary bifurcation and blockage of the drainage of the contralateral liver lobe.

Choice of stent, plastic or metal, has largely remained at the discretion of the endoscopist and patient. In this systematic review and meta-analysis (SRMA), we aimed to compare the outcomes of management of BBS with the use of multiple plastic stents (MPS) and cSEMS.

## Review

Materials and methods

This systematic review and meta-analysis was performed in accordance with the Preferred Reporting Items for Systematic Reviews and Meta‐Analyses (PRISMA) guidelines and registered with the PROSPERO (CRD42021289002).

Information Sources and Search Strategy

We searched MEDLINE, Cochrane Central Register of Controlled Trials (CENTRAL), and Science Direct from 2000 to September 2021 for all relevant studies. A search was made using the keywords: "Benign bile duct stricture" OR "anastomotic bile duct stricture" OR "Biliary stricture" OR "Chronic pancreatitis related bile duct stricture" AND "self-expandable metallic stent" OR "SEMS" OR "metal stent" OR "plastic stents" OR "Multiple plastic stent." Additionally, we searched the reference lists of all identified trials, guidelines, and reviews on the topic for relevant trials.

Study Selection

Two independent reviewers searched the titles and abstracts of the retrieved search records for inclusion and exclusion criteria. The same two reviewers examined the full text of potential eligible citations. Any disagreement was resolved by a third reviewer. Studies included in this SRMA were randomized controlled trials (RCTs) fulfilling the following PICO criteria: (a) patients - BBS associated with CP, or bile duct injury and post-LT anastomotic strictures; (b) intervention - placement of cSEMS for BBS; (c) comparison - placement of MPS for BBS; (d) outcomes - stricture resolution, number of sessions of ERCP for stricture resolution, recurrence of stricture, stent-migration, moderate-severe adverse events, and cost-analysis. We included only original articles and conference abstracts were excluded. There was no bar on language as long as study outcomes are mentioned in the text. Non-randomized studies, case series with sample size < 10, case series, and studies involving persons < 18 years of age were excluded from the analysis.

Outcomes Assessed

Stricture resolution was defined on the basis of cholangiogram showing easy passage of contrast across the stricture during ERCP at the end of endoscopic treatment and improvements in clinical and liver function test. Stricture recurrence was defined by cholangiographic evidence of biliary stricture among patients who had prior resolution of stricture and the need for reintervention during the follow-up period after initial resolution. Moderate-severe adverse events included pancreatitis, cholangitis, cholecystitis, perforation, hemorrhage, severe pain requiring admission, and infection. Cost-analysis was performed taking into account the cost of the procedure, hospital stay, and all the accessories used.

Data Extraction

Data extraction was performed independently by two investigators. Any disagreement was resolved by a third reviewer. Data collection was done under the following headings: study author and year, type of stricture, number of patients, sex distribution, type of intervention used and the comparator arm, follow-up duration, outcomes, and adverse events.

Risk of Bias in Individual Studies and Confidence in Cumulative Evidence

The risk of bias was assessed by two reviewers using the Cochrane risk-of-bias tool for randomized trials (RoB 2) [7]. The quality of evidence was analyzed according to the Grading of Recommendations Assessment, Development and Evaluation Working Group (GRADE) approach [8].

Statistical Analysis

Hozo's formula was used to convert medians and ranges into mean and standard deviations [9]. Dichotomous variables were analyzed using risk ratio and Mantel-Haenszel test, whereas continuous variables were analyzed using mean difference and inverse variance. The random-effect model was used irrespective of the presence of heterogeneity. The Q statistic test and I^2^ statistics were used for the assessment of heterogeneity among the studies. A p-value of Q test < 0.1 or the I^2^ value > 50% was considered to be significant. The assessment of publication bias was done using funnel plots and Egger’s test. All statistical analyses were performed using RevMan software version 5.4.1 (Cochrane Collaboration) and STATA software version 17 (College Station, TX: StataCorp.).

Results

**Figure 1:**
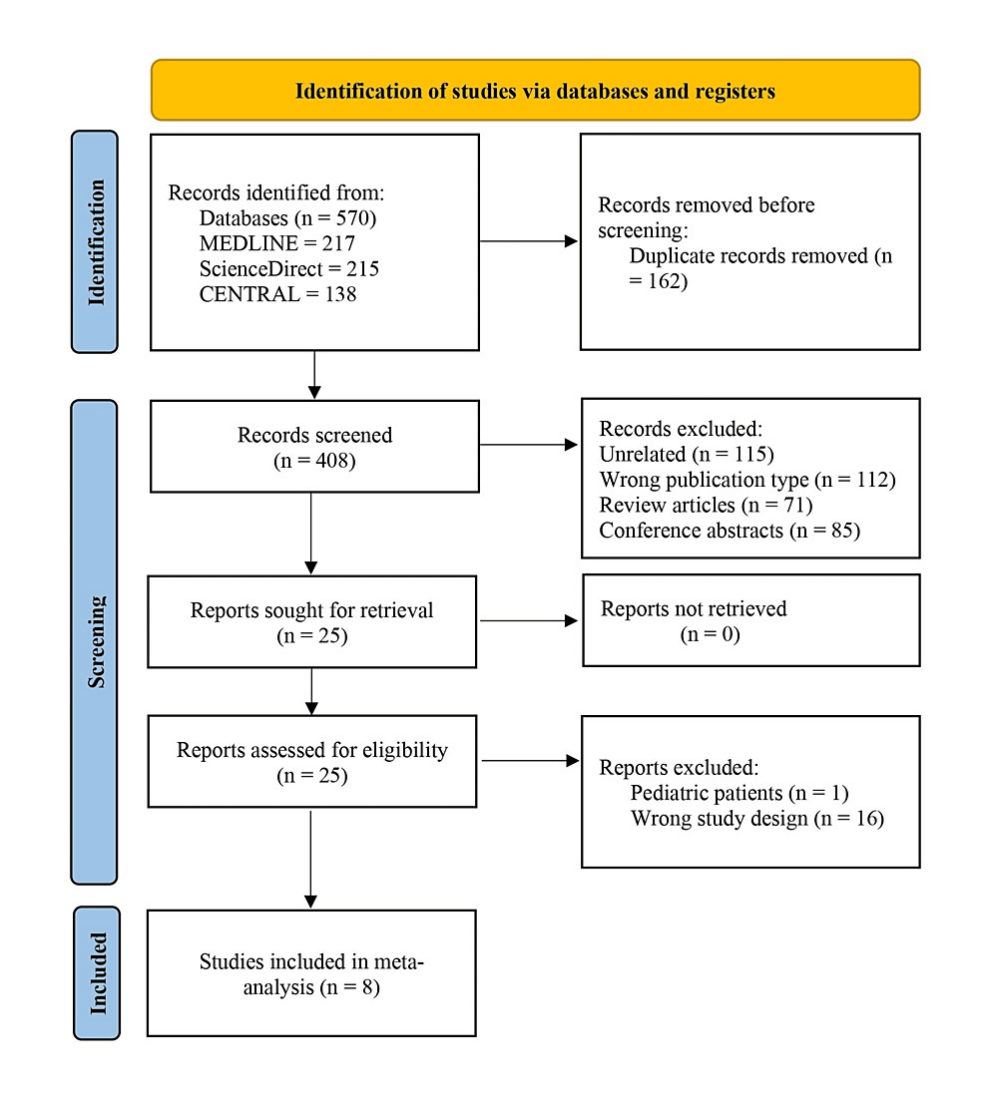
Flow diagram for study retrieval and identification for meta‐analysis as per the PRISMA 2020 statements PRISMA: Preferred Reporting Items for Systematic Reviews and Meta‐Analyses

Figure 1 shows the PRISMA flowchart for the article selection process which was conducted as per the updated guidelines (table in Appendix) [10]. Finally, eight RCTs were included in the analysis [11-18].

Table 1 summarizes the characteristics of the study population and their outcomes. Among the studies, one study included patients with bile duct injury [11], two studies included BBS due to CP [13,18], three studies included patients with post-LT anastomotic stricture [12,15-17], and one study included mixed etiologies [14]. The duration of stent placement ranged from three to 12 months for cSEMS and the interval to replace the plastic stents ranged from six to 16 weeks. 

**Table 1:** Characteristics of included studies *AS/CP/BI: 31/8/2. **AS/CP/BI: 33/15/2. AS: anastomotic stricture; CP: chronic pancreatitis; BI: bile duct injury; ERCP: endoscopic retrograde cholangiopancreatography; MPS: multiple plastic stents; cSEMS: covered self-expandable metallic stents

Author	Country	Arm	No. of patients	Sex (M/F)	Age, years	Etiology (AS/CP/BI)	ERCP sessions	Success rate	Recurrence	Stent migration	Adverse events	Follow-up time, months
Artifon et al., 2012 [11]	Brazil	MPS	16	6/10	45.19	0/0/16	-	16	5	2	4	72
		cSEMS	15	5/10	45.53	0/0/15	-	15	3	0	3	72
Kaffes et al., 2014 [12]	Australia	MPS	10	5/5	49.5 (23-69)	10/0/0	4.0 ± 1.17	8	3/8	1	5	25.5 (3-44)
		cSEMS	10	5/5	56.5 (38-67)	10/0/0	2.0 ± 0.20	10	3/10	0	1	26 (6-40)
Haapamaki et al., 2015 [13]	Finland	MPS	30	29/1	49.5 (30-69)	0/30/0	-	22	3/22	3	7	37 (3-61)
		cSEMS	30	25/5	54.5 (30-78)	0/30/0	-	20	2/20	2	8	41 (1-66)
Cote et al., 2016 [14]	USA	MPS	55	37/17	56.7 ± 11	36/17/2	3.13 ± 0.88	41/48*	2/41	9	11	24
		cSEMS	57	38/19	54.5 ± 10.4	37/18/2	2.21 ± 0.48	50/54**	7/50	14	11	24
Martins et al., 2018 [15]	Brazil	MPS	29	20/9	50 (28-71)	29/0/0	4.9 ± 0.60	28	0/28	4	4	32.9
		cSEMS	30	22/8	54 (23-73)	30/0/0	2.0 ± 0.20	25	8/25	3	12	36.4
Tal et al., 2017 [16]	Europe	MPS	24	18/6	58.5 (32-72)	24/0/0	5.75 ± 2.61	23	5/23	0	2	16.9 (2-39.4)
		cSEMS	24	14/10	57 (32-69)	24/0/0	2.0 ± 0.20	24	5/24	8	0	13.3 (6.3-34.9)
Cantu et al., 2021 [17]	Italy	MPS	15	14/1	53 (22-68)	15/0/0	4.5 ± 1.15	14	1/14	2	6	10 (4-24)
		cSEMS	15	12/3	59 (50-67)	15/0/0	4.0 ± 1.76	11	4/11	5	3	9 (4-26)
Ramchandani et al., 2021 [18]	Multicenter	MPS	84	72/12	53 (26-74)	0/84/0	3.9 ± 1.3	54/70	-	18/82	16/82	24
		cSEMS	80	70/10	51 (28-74)	0/80/0	2.6 ± 1.3	47/62	-	15/80	19/80	24

Rate of Stricture Resolution

**Figure 2:**
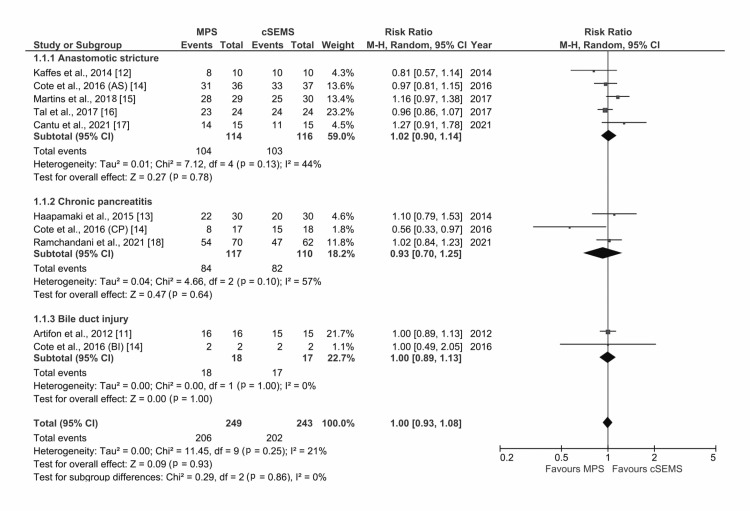
Forest plot comparing cSEMS and MPS for benign biliary stricture resolution with subgroup analysis based on etiology of stricture MPS: multiple plastic stents; cSEMS: covered self-expandable metallic stents

All the eight RCTs reported data on the rate of stricture resolution [11-18]. The analysis of the forest plot showed similar rate of resolution of BBS with MPS and cSEMS (risk ratio {RR}: 1.00, 95% confidence interval {CI}: 0.89-1.08; I2 = 21%, p=1.00) with low heterogeneity (Figure 2). On subgroup analysis, there was no difference in the rate of stricture resolution with use of MPS or cSEMS in those with anastomotic stricture, stricture due to chronic pancreatitis, or bile duct injury.

Number of ERCP Sessions

**Figure 3:**
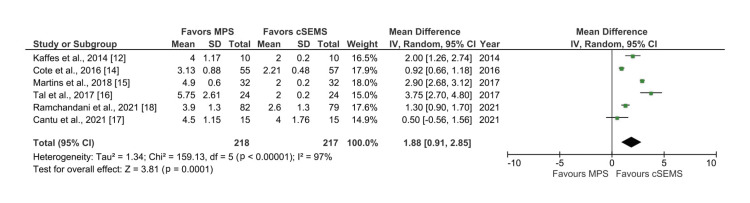
Forest plot comparing cSEMS and MPS for number of ERCP sessions required for stricture resolution ERCP: endoscopic retrograde cholangiopancreatography; MPS: multiple plastic stents; cSEMS: covered self-expandable metallic stents

Overall, six studies reported the data on number of sessions required for stricture resolution [12,14-18]. Analysis of the forest plot showed significantly lower number of ERCP sessions with the use of cSEMS (mean difference: 1.88, 95% CI: 0.91-2.85; I2 = 97%, p < 0.00001), although there was significant heterogeneity among the studies (Figure 3).

Recurrence of Stricture

**Figure 4:**
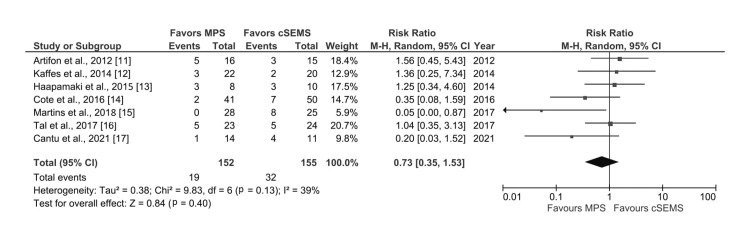
Forest plot comparing cSEMS and MPS for recurrence of stricture after resolution MPS: multiple plastic stents; cSEMS: covered self-expandable metallic stents

The data on the rate of stricture recurrence on follow-up after resolution was reported by seven studies [11-17]. The recurrence rate of BBS was comparable between both MPS and cSEMS group (RR: 0.73, 95% CI: 0.35-1.53; I2 = 39%, p=0.13) with moderate level of heterogeneity (Figure 4).

Stent Migration

**Figure 5:**
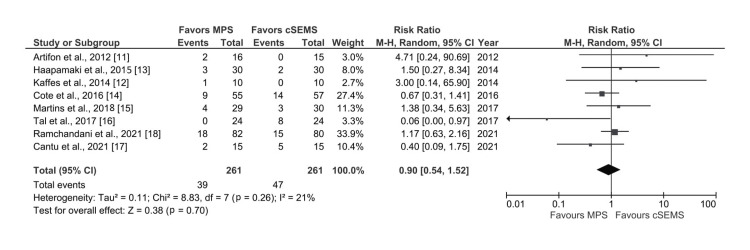
Forest plot comparing cSEMS and MPS for rate of stent migration MPS: multiple plastic stents; cSEMS: covered self-expandable metallic stents

All eight studies reported data on the incidence of stent migration in the patients [11-18]. There was no significant difference between both groups with respect to rate of stent migration (RR: 0.90, 95% CI: 0.54-1.52; I^2^ = 21%, p=0.26) with low heterogeneity (Figure 5).

Moderate-Severe Adverse Events

**Figure 6:**
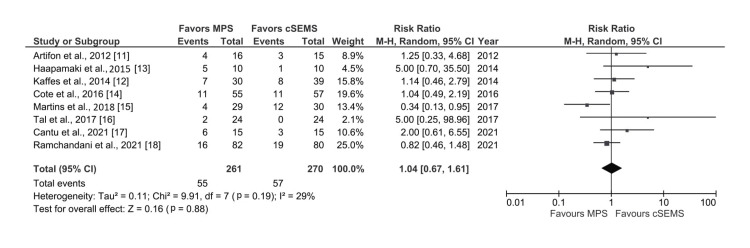
Forest plot comparing risk of adverse events with cSEMS and MPS MPS: multiple plastic stents; cSEMS: covered self-expandable metallic stents

All eight studies reported data on moderate-severe adverse events which included pancreatitis, hemobilia, severe pain abdomen requiring admission, perforation, acute bacterial cholangitis, acute cholecystitis, infection of pseudocyst, duodenal obstruction, and bleeding from pseudocyst [11-18]. However, there was no significant difference in the rate of adverse events in both groups (RR: 1.04, 95% CI: 0.67-1.61; I^2^ = 29%, p=0.19) with low heterogeneity (Figure 6).

Risk of Bias

**Figure 7:**
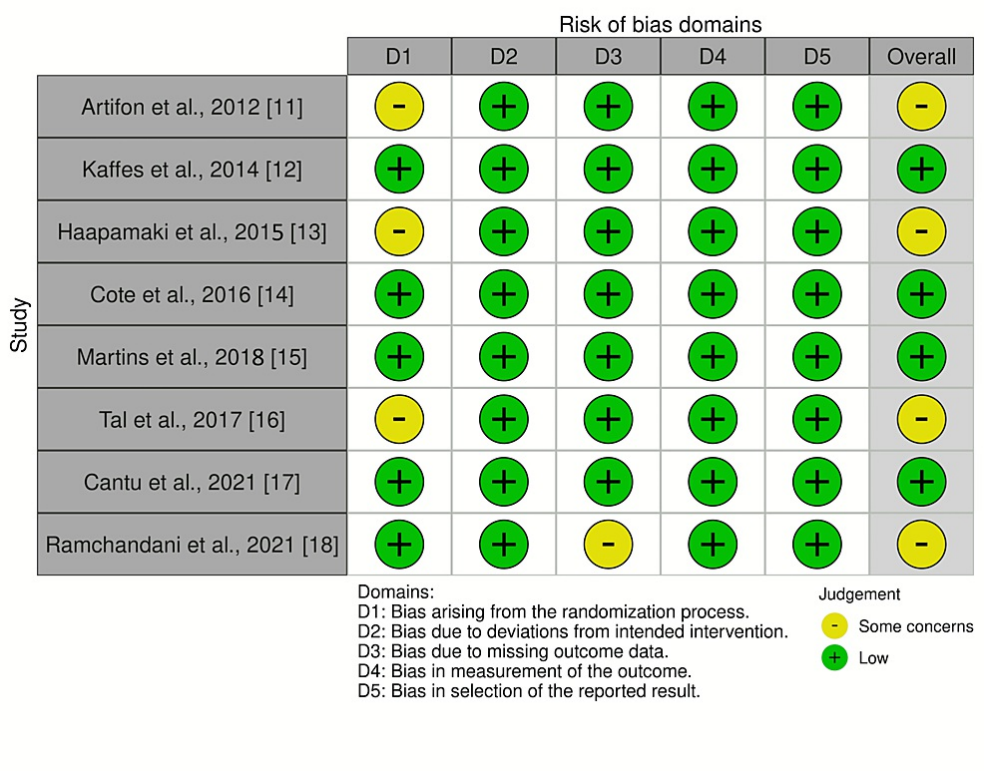
Traffic light plot for risk of bias in randomized controlled trials

Among the studies, four studies had no risk of bias while four other studies had some concern with regards to the risk of bias [11-18]. The traffic-light plot for risk of bias are shown in Figure 7.

Publication Bias and Grade of Evidence

**Figure 8:**
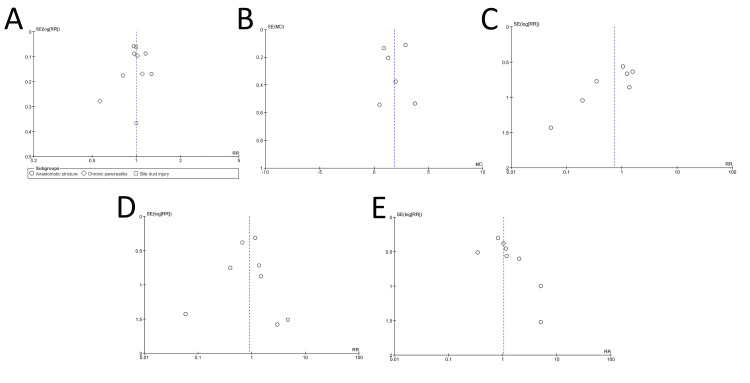
Funnel plots for assessment of publication bias for outcomes The image shows (A) stricture resolution, (B) number of sessions of ERCP, (C) recurrence of stricture, (D) stent migration, and (E) moderate-severe adverse events. ERCP: endoscopic retrograde cholangiopancreatography; RR: risk ratio

Visual assessment of the funnel plots (Figures 8A-8E) showed asymmetry in the plots for stricture recurrence (Figure 8C) and moderate-severe adverse events (Figure 8E). Egger’s test for all the outcomes showed evidence of publication bias only for stricture recurrence (Table 2). Table 3 shows the summary of findings with the grade of evidence. 

**Table 2:** Egger's test for assessment of small study effect for various outcomes ERCP: endoscopic retrograde cholangiopancreatography; CI: confidence interval

Outcome	Coefficient	Std. error	t	P > t	95% CI	
Stricture resolution	0.9937121	1.118807	0.89	0.409	-1.74391	3.731335
Number of ERCP sessions	-1.417426	4.968966	-0.29	0.79	-15.21349	12.37863
Stricture recurrence	-3.489618	1.108748	-3.15	0.025	-6.33974	-0.63949
Stent migration	-0.0845657	0.8104068	-0.1	0.920	-2.06756	1.898428
Moderate-severe adverse events	1.643596	0.9218253	1.78	0.125	-0.612029	3.899221

**Table 3:** Summary of findings (population - benign biliary stricture; intervention - multiple plastic stents; comparison - covered self-expanding metal stent) AE: adverse events; CI: confidence interval; cSEMS: covered self-expandible metallic stents; ERCP: endoscopic retrograde cholangiopancreatography; MPS: multiple plastic stents

Outcomes	Anticipated absolute effects (95% CI)		Relative effect (95% CI)	No. of participants (studies)	Certainty assessment				Overall certainty of evidence
	Risk with MPS	Risk with cSEMS			Risk of bias	Inconsistency	Indirectness	Imprecision	
Stricture resolution	827 per 1000	831 per 1000 (757 to 907)	RR 1.00 (0.93-1.08)	492 (8 studies)	+	-	-	-	Moderate ●●●○
No. of ERCP sessions	Mean no. of sessions = 4.10	1.88 lower (0.91 to 2.85)	MD 1.88 (0.91 to 2.85)	435 (6 studies)	+	+	-	-	Low ●●○○
Stricture recurrence	125 per 1000	205 per 1000 (85 to 325)	RR 0.73 (0.35-1.53)	307 (7 studies)	+	-	-	+	Low ●●○○
Stent migration	150 per 1000	180 per 1000 (80 to 270)	RR 0.90 (0.54-1.52)	522 (8 studies)	+	-	-	-	Moderate ●●●○
Moderate-severe AE	210 per 1000	190 per 1000 (90 to 290)	RR 1.04 (0.67-1.61)	531 (8 studies)	+	-	-	-	Moderate ●●●○

Discussion

Previous meta-analyses have reported cSEMS to be comparable to MPS in terms of stricture resolution rate [19-21]. Our meta-analysis further strengthens this evidence and unlike previous meta-analysis primarily compares both modes of endoscopic therapy; also provides subgroup analysis on basis of etiology for initial treatment success [19-21]. This is important as biliary strictures secondary to chronic pancreatitis are usually difficult to treat due to associated fibrosis and calcification [18]. Despite comparable stricture resolution rates, cSEMS required significantly lesser number of ERCP sessions as per our analysis. This was expected as most patients with cSEMS typically require only two ERCP sessions (one for insertion and other for removal) whereas patients with MPS require serial exchange of stents every three months usually for one year. However, whether lesser ERCP sessions for initial stricture resolution translates into lesser cost and better long-term outcomes also depends upon other factors like stricture recurrence and adverse effects apart from the cost of accessories.

Our analysis reported that stricture recurrence rate was not statistically different among cSEMS and MPS. This is consistent with previous meta-analysis and several RCTs [12,14,16,19,21]. However, recent RCT by Cantu et al. in the post liver transplant patients reported stricture recurrence rate of 36% in cSEMS and 7% in MPS patients (p = NS), and re-treatment was needed in 53% and 13% (p < 0.01), respectively, during follow-up of 60 (34-80) months [17]. Similarly, Martin et al. reported recurrence rate of 32% in cSEMS vs 0% in MPS group in post liver transplant anastomotic stricture after average follow-up of approximately three years [15]. They proposed a short indwelling duration of cSEMS (median duration of six months) as likely explanation for this difference. Meta-analysis by Khan et al. reported a significant inverse relationship between the duration of stent therapy and stricture recurrence rates especially, in post-surgery and liver transplant patients [21]. Ramchandani et al. used longer indwelling time for cSEMS and compared 12-month treatment with MPS vs cSEMS for symptomatic CP-associated BBS in a recent RCT and reported that stricture resolution status at 24 months was 77.1% (54/70) vs 75.8% (47/62) (p = 0.008 for noninferiority intention-to-treat analysis), respectively [18]. Prospective multinational studies by Tringali et al. and Lakhtakia et al. reported good long-term outcomes after temporary placement of cSEMS (10-12 months indwelling time) in post-cholecystectomy (non-hilar) and CP-related BBS, respectively [22,23].

Theoretically, cSEMS appear to have greater risk of stent migration and this is an area of concern as it can affect long-term outcomes of endo-therapy. Risk of migration remains with MPS too in view of associated sphincterotomy. Our analysis found no statistically significant difference in stent migration rates between MPS and cSEMS. A different type of prosthesis used in some RCTs may prevent generalization on this topic. A recent meta-analysis by Yang et al. on efficacy of different endoscopic stents in the management of post-operative biliary strictures reported modified cSEMS with antimigration waist or a cone shape to reduce stent migration, is more favorable in the management of BBS compared with MPS or conventional cSEMS [24]. However, two recent RCTs published in 2021 requires special mention in view of conflicting results in some of the previous RCTs on this topic. Cantu et al. analyzed patients with post liver transplant anastomotic stricture and found high stent migration rates with fully covered, self-expanding metal stent (FCSEMS) as compared to MPS (29% vs 2.6%, p=<0.01) especially when used as the first-line therapy [17]. Also need for retreatment was significantly higher in migrated FCSEMS group. Ramchandani et al. in an analysis of CP-related BBS reported approximately 20% stent migration in both MPS and FCSEMS groups [18]. This shows factors other than peri stricture scarring (as seen in CP-related BBS) control migration of stent. A recent randomized controlled trial analyzed the benefit of an internal anchoring double pigtail plastic stent in patients with malignant distal biliary obstruction managed using cSEMS versus cSEMS alone [25]. There was a significantly lower rate of migration at six months (15% vs 40%, p=0.02) with longer mean stent patency (237 days vs 173 days, p=0.048). Further RCTs are needed to compare cSEMS with antimigration properties or indwelling plastic stents with MPS on different populations of BBS.

Our analysis found no statistically significant difference in the rate of moderate-severe adverse effects in both groups. Martin et al. in their RCT reported high acute pancreatitis rates in cSEMS group as compared to MPS group (13.1% vs 2.1 %) which reduced drastically after performing sphincterotomy in cSEMS group [15]. Another area of concern remaining after the above discussion is cost-effectiveness of each approach. Though insufficient data precluded detailed cost analysis in our meta-analysis, recent evidence on this aspect needs to be highlighted. Previously, two RCTs [12,15] and a meta-analysis [20] (calculated average cost from both these RCTs) reported cSEMS to be a more cost-effective option as compared to MPS. However, there was heterogeneity in both these RCTs in terms of cost analysis as Martin et al. did not include cost of hospital stay and re-treatment in their analysis [15]. Jang et al. reported transition to FCSEMS at the second ERCP (after index ERCP with PS) could provide 25% reduction in total procedure cost to achieve anastomotic biliary stricture resolution [26]. Cantu et al. in their cost analysis RCT with long-term follow up as mentioned previously, reported suboptimal performance of cSEMS as first-line treatment for biliary anastomotic stricture after liver transplantation due to higher stent migration rate and need for re-treatment [17]. Another hypothesis given in this study was regarding sudden expansion of metal flanges in anastomotic stricture causing ischemic damage as compared to a slower and controlled expansion with MPS [17]. However, the use of cSEMS in patients in clinical remission after either cSEMS or MPS was associated with reduction in cost of up to 40%. Long-term follow-up in this RCT (more than 34 months with a median follow-up duration of five years after the end of endoscopic therapy) ensured the inclusion of any retreatment-related cost. Small sample size was a major limitation of this study.

Our meta-analysis included the largest number of RCTs including the two RCTs from 2021 with long-term outcomes on the efficacy and safety of cSEMS for the management of BBS in comparison to the deployment of multiple plastic stents. This is the first meta-analysis with subgroup analysis based on etiology of BBS for initial treatment success, which is important, as CP-related BBS are difficult to treat as compared to post-operative BBS for reasons described above. We analyzed the available literature and systematic review of relevant studies for long-term cost-effectiveness of both approaches, which can have a significant impact on the choice of stent. This meta-analysis had few limitations. There is considerable heterogeneity in results of our primary analysis in view of variable duration of indwelling stent, different type of stents used, and inclusion of BBS with different etiologies. However, random-effects model was used for all outcomes to get more conservative estimates. Meta-regression analysis was not possible to study the effect of these variables on outcomes, as we have included less than 10 studies in our analysis. Only a limited number of RCTs reported cost analysis data so detailed cost-effectiveness was not possible for both modes of therapy in our analysis, however, we provided a brief overview of available data from RCTs and prospective studies.

## Conclusions

In the light of growing evidence comparing MPS versus cSEMS for the treatment of BBS, no definitive conclusions can be drawn regarding the absolute superiority of one over the other. cSEMS is comparable to MPS for management of BBS in terms of stricture resolution rates with the requirement of lesser number of ERCP sessions irrespective of etiology with similar adverse effects. The use of conventional cSEMS as the first-line options in post-operative strictures is still a matter of debate as per recent evidence and requires long-term RCTs with adequate sample size comparing cSEMS with anti-migration features with MPS technique. cSEMS can be used as the first-line option in CP-related BBS. Additional research into the cost-effectiveness of the two strategies for each type of BBS (based on etiology) would also help clinicians deciding optimal treatment approach.

## Appendices

**Table 4:** PRISMA checklist for systematic review and meta-analysis PRISMA: Preferred Reporting Items for Systematic Reviews and Meta‐Analyses

Section and topic	Item #	Checklist item	Location where item is reported
Title			
Title	1	Identify the report as a systematic review.	1
Abstract			
Abstract	2	See the PRISMA 2020 for Abstracts checklist.	2
Introduction			
Rationale	3	In light of existing knowledge, explain the rationale behind the review.	3
Objectives	4	Explain explicitly what the review's objectives or questions are.	3
Methods			
Eligibility criteria	5	Outline the inclusion and exclusion criteria for the review and the division of studies for the syntheses.	4
Information sources	6	Specify all databases, registers, websites, organisations, reference lists and other sources searched or consulted to identify studies. Specify the date when each source was last searched or consulted.	4
Search strategy	7	Include all search strategies, filters and limits used for all databases, registers and websites.	4
Selection process	8	Details of how each record and each report retrieved was reviewed, how many reviewers performed a review, and whether the reviewers worked independently, as well as the use of automation tools, if any, to be specified.	4
Data collection process	9	Indicate the methods used to collect data from reports, including the number of reviewers who collected data from each report, whether they worked independently, any processes for obtaining or correlating information from researchers, and if applicable, the types of automation tools used to complete the process.	4
Data items	10a	List and define all outcomes for which data were sought. Specify whether all results that were compatible with each outcome domain in each study were sought (e.g. for all measures, time points, analyses), and if not, the methods used to decide which results to collect.	4
	10b	List and define all other variables for which data were sought (e.g. participant and intervention characteristics, funding sources). Describe any assumptions made about any missing or unclear information.	4
Study risk of bias assessment	11	Specify the methods used to assess risk of bias in the included studies, including details of the tool(s) used, how many reviewers assessed each study and whether they worked independently, and if applicable, details of automation tools used in the process.	4
Effect measures	12	Specify for each outcome the effect measure(s) used in the synthesis or presentation of results.	5
Synthesis methods	13a	Describe the processes used to decide which studies were eligible for each synthesis	5
	13b	Methods of preparing the data to be presented or synthesized, such as the addition of missing summary statistics or the conversion of data, should be described..	5
	13c	Describe the methods used for tabulation or displaying the results of individual studies and syntheses.	5
	13d	Describe any methods used to synthesize results and provide a rationale for the choice(s). If meta-analysis was performed, describe the model(s), method(s) to identify the presence and extent of statistical heterogeneity, and software package(s) used.	5
	13e	Methods used for assessment of heterogeneity	5
	13f	Assess robustness of the meta-analysis by sensitivity analyses	5
Reporting bias assessment	14	Describe any methods used to assess risk of bias due to missing results in a synthesis (arising from reporting biases).	4
Certainty assessment	15	Describe any methods used to assess certainty (or confidence) in the body of evidence for an outcome.	4
Results			
Study selection	16a	Describe the results of the search and selection process, from the number of records identified in the search to the number of studies included in the review, ideally using a flow diagram.	5
	16b	Explain why studies that seemed to fit the inclusion criteria were excluded, and provide examples.	5
Study characteristics	17	Cite each included study and present its characteristics.	Table 1
Risk of bias in studies	18	Risk of bias assessment for each included study.	5
Results of individual studies	19	For all outcomes, present, for each study: (a) summary statistics for each group (where appropriate) and (b) an effect estimate and its precision (e.g. confidence/credible interval), ideally using structured tables or plots.	Table 1
Results of syntheses	20a	Briefly summarize the characteristics and bias risks among the contributing studies for each synthesis.	5
	20b	Present results of all statistical syntheses conducted. If meta-analysis was done, present for each the summary estimate and its precision (e.g. confidence/credible interval) and measures of statistical heterogeneity. If comparing groups, describe the direction of the effect.	5, 6
	20c	Describe the possible causes of heterogeneity among study results.	5, 6
	20d	Assessment of robustness of the synthesized results by sensitivity analyses.	6
Reporting biases	21	Present assessments of risk of bias due to missing results (arising from reporting biases) for each synthesis assessed.	Supplementary Figure 1
Certainty of evidence	22	Present assessments of certainty (or confidence) in the body of evidence for each outcome assessed.	Table 2
Discussion			
Discussion	23a	In light of other evidence, explain your interpretation of the results.	6, 7
	23b	Review the evidence and discuss any limitations.	9
	23c	The limitations of the review process should be discussed.	9
	23d	Explain future research, practice, and policy implications of the results.	8
Other information			
Registration and protocol	24a	Provide registration information for the review, including register name and registration number, or state that the review was not registered.	3
	24b	Provide a link to the review protocol, or mention that a protocol has not been prepared.	PROSPERO
	24c	Any changes to the information provided at registration or in the protocol should be described and explained.	N/A
Support	25	Describe how the review was supported financially or non-financially, and who funded or sponsored it.	1
Competing interests	26	Declare any competing interests of review authors.	1
Availability of data, code and other materials	27	Report which of the following are publicly available and where they can be found: template data collection forms; data extracted from included studies; data used for all analyses; analytic code; any other materials used in the review.	N/A
